# A grazing *Gomphotherium* in Middle Miocene Central Asia, 10 million years prior to the origin of the Elephantidae

**DOI:** 10.1038/s41598-018-25909-4

**Published:** 2018-05-16

**Authors:** Yan Wu, Tao Deng, Yaowu Hu, Jiao Ma, Xinying Zhou, Limi Mao, Hanwen Zhang, Jie Ye, Shi-Qi Wang

**Affiliations:** 1 Key Laboratory of Vertebrate Evolution and Human Origins of Chinese Academy of Sciences, Institute of Vertebrate Paleontology and Paleoanthropology, Chinese Academy of Sciences, Beijing, 100044 China; 2 CAS Center for Excellence in Life and Paleoenvironment, Beijing, 100044 China; 3 CAS Center for Excellence in Tibetan Plateau Earth Sciences, Beijing, 100101 China; 4 Key Laboratory of Economic Stratigraphy and Palaeogeography, Nanjing Institute of Geology and Palaeontology, Chinese Academy of Sciences, Nanjing, 210008 China; 5 School of Earth Sciences, University of Bristol, Life Sciences Building, 24 Tyndall Avenue, Bristol, BS8 1TQ UK; 6 Earth Sciences Department, Natural History Museum, Cromwell Road, London, SW7 5BD UK; 7 Department of Archaeology and Anthropology, University of Chinese Academy of Sciences, Beijing, 100049 China

## Abstract

Feeding preference of fossil herbivorous mammals, concerning the coevolution of mammalian and floral ecosystems, has become of key research interest. In this paper, phytoliths in dental calculus from two gomphotheriid proboscideans of the middle Miocene Junggar Basin, Central Asia, have been identified, suggesting that *Gomphotherium connexum* was a mixed feeder, while the phytoliths from *G*. *steinheimense* indicates grazing preference. This is the earliest-known proboscidean with a predominantly grazing habit. These results are further confirmed by microwear and isotope analyses. Pollen record reveals an open steppic environment with few trees, indicating an early aridity phase in the Asian interior during the Mid-Miocene Climate Optimum, which might urge a diet remodeling of *G*. *steinheimense*. Morphological and cladistic analyses show that *G*. *steinheimense* comprises the sister taxon of tetralophodont gomphotheres, which were believed to be the general ancestral stock of derived “true elephantids”; whereas *G*. *connexum* represents a more conservative lineage in both feeding behavior and tooth morphology, which subsequently became completely extinct. Therefore, grazing by *G*. *steinheimense* may have acted as a behavior preadaptive for aridity, and allowing its lineage evolving new morphological features for surviving later in time. This study displays an interesting example of behavioral adaptation prior to morphological modification.

## Introduction

The coevolution of plants and mammals amid dramatic Cenozoic climactic perturbations has been the focus of intensive research^1,2^. There is now abundant evidence to suggest that grassland ecosystems dominated by the family Gramineae expanded markely in the Neogene, and promoted significant adaptive evolutionary responses in various herbivorous mammal lineages^2^. For example, proboscideans, the largest Neogene herbivores which were ‘super-keystone species’ in their ecosystems^3^, modified their grinding teeth from low-crowned bunolophodont (“mastodont-like”) molars with few loph(id)s (enamel cones fused in a line), to high-crowned and multi-plated (“elephant-like”) teeth allowing them to consume more abrasive foods^4^. At least for proboscideans, several analytical approaches have been adopted to track this process, such as enamel microwear^3,5^ and stable isotope studies^6^. However, any single given palaeodietary proxy *per se* is insufficient to provide extensively detailed inferences about the feeding ecology of fossil organisms. Therefore, a multi-proxy approach is essential for performing palaeodietary investigations.

Dental calculus is the calcium phosphate deposited on teeth, and captures a large number of food particles that provide crucial information about the food of ancient animals^7,8^. Phytoliths sealed within the calculus, in particular, provide an extensive dietary record^9–11^. Due to their strong resistance to decay and to mechanical or biological decomposition, phytoliths can be well preserved for a long time^12^. Phytoliths have been extracted from long-dead animals that span a great temporal scope, from ancient humans^8^ to an Early Cretaceous dinosaur^13^. A wide range of phytoliths have also recently been recovered from the dental calculus of Pleistocene proboscideans, such as *Notiomastodon*^14^ and *Mammut*^15^. Because of their consistent shape within different plant taxa, phytoliths therefore provide a very strong taxonomic signal^12,16^. With diagnosability to the family, genus or even species level, phytoliths thus provide a reliable basis to explore the feeding ecology and palaeoenvironments of ancient animals, beyond the level of resolution available from other palaeodietary proxies such as dental microwear and stable isotopes, which generally provide broad ‘C3/C4’ inferences (which themselves act as proxy for ‘browsing/grazing’) for herbivores, although these proxies provide useful complementary data^17^.

**Figure 1:**
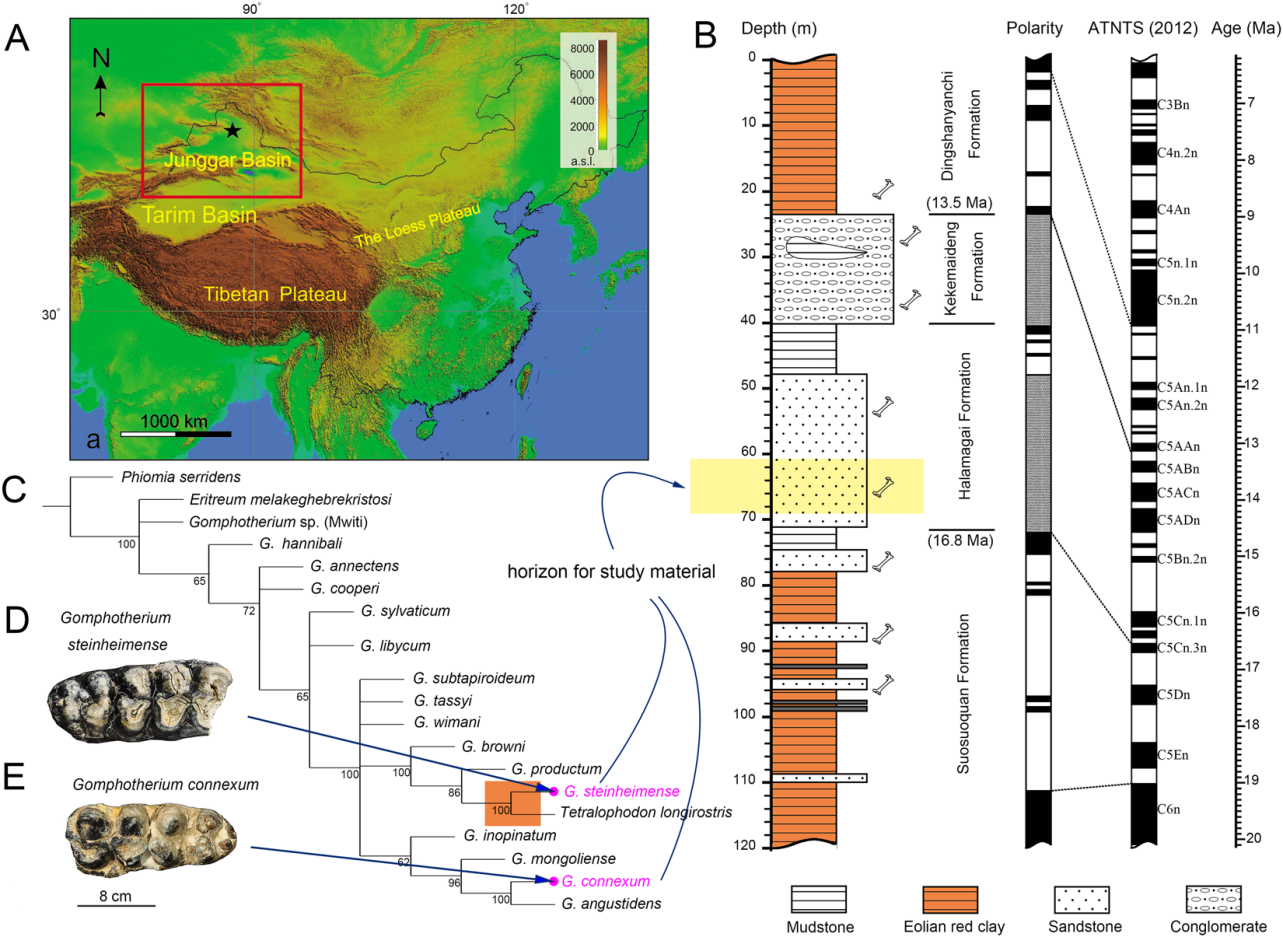
Geography, geology, and phylogeny in relation to the study material. (**A**) The location of the study area (black star), details are shown in Fig. S1. The map was generated by GTOPO309 (http://www1.gsi.go.jp/geowww/globalmap-gsi/gtopo30/gtopo30.html) using Globalmapper (v10) (http://www.bluemarblegeo.com/products/global-mapper.php). (**B**) Stratigraphic column and polarity with palaeomagnetic age (redrawn after refs^18,19^), also denoting the horizon of study material in the strata (in light yellow). (**C**) The 50% majority consensus tree from 29 maximum parsimonious trees showing the phylogenetic position of the *Gomphotherium* species and *Tetralophodon longirostris* (tree length = 81, CI = 0.617; RI = 0.744, data set in Table S1), the number at each node representing the support value calculated by majority rules (percentages of supported MPTs in the total MPTs, which are always larger than 50%) and the orange frame indicating the sister-taxon relationship of *G*. *steinheimense* and *T*. *longirostris*. (**D**) *G*. *steinheimense*, IVPP V23283, right m3. (**E**) *G*. *connexum*, IVPP V8576, left M3.

In the present article, we examine the feeding ecology of two species of gomphotheriid proboscideans, *Gomphotherium connexum* and *G*. *steinheimense*, based on phytoliths from their dental calculus, complemented by dental stereomicrowear and stable isotopic data. Our studied samples are discovered from the Miocene Halamagai Formation, northern Junggar Basin, Xinjiang, China (Figs 1, S1). Previous studies and our pollen data have shown that the Halamagai Formation covers the Mid-Miocene Climate Optimum (MMCO)^18^ and records the major palaeoenvironmental perturbation crises from a more humid Middle Miocene environment to the comparatively more arid Late Miocene ecosystem^19,20^ (SI 1.1). This palaeoenvironmental transition had a profound impact on the evolution of terrestrial floras and faunas in Central Asia and elsewhere. Our gomphothere dietary reconstruction indicates the disparate feeding habits of the two *Gomphotherium* species from Halamagai Formation. Furthermore, cladistic analysis also indicates the distinct phylogenetic affinities of the two species (Fig. 1C, SI 1.2, 1.3). Therefore, our research is of importance not only in understanding palaeoenvironmental changes in the middle-latitude zone of inland Asia during the Middle to Late Miocene, but also holds broader significance in understanding the coevolution of plant communities and mammals in terms of habitats and feeding ecology.

## Results

### Phytoliths analysis

**Figure 2:**
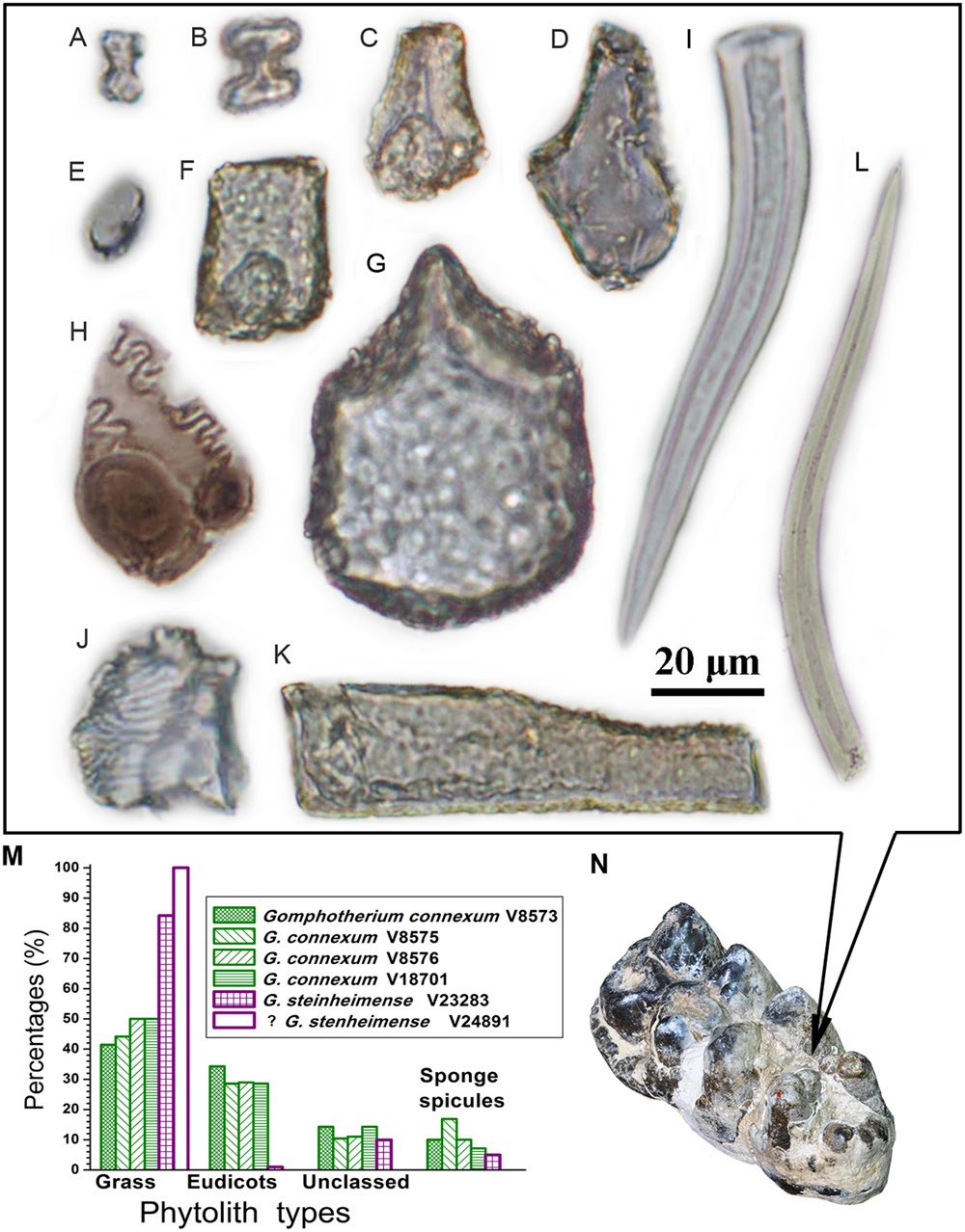
Phytolith and sponge spicules, as well as their statistics in *Gomphotherium* dental calculus. (**A**–**L**) Morphology of various phytolith, including bilobates short cell (V8573) (**A**), bilobates short cell (V23283) (**B**), bulliform (V8575) (**C**), bulliform (V23283) (**D**), rondel (V23283) (**E**), rectangular (V8576) (**F**), reed-type bulliform (V23283) (**G**), long cell (V8575) (**H**), hair cell (V8576) (**I**), irregular multifaceted (V8573) (**J**), elongate (V8575) (**K**) sponge spicules (V8576) (**L**). (**M**) Percentages of grass phytoliths, eudicots phytoliths, unclassed phytoliths and sponge spicules occurring in *Gomphotherium* dental calculus. (**N**) Example of dental calculus sampling (the tooth is V8576).

Our method for extracting phytoliths from dental calculus was successful for four molars of *G*. *connexum* and two of *G*. *steinheimense* (see the Materials and Methods section). The samples contain grass phytolith morphotypes including several types of short cells and long cells (Table 1 and Fig. 2A–K). The main forms of grass short cell phytoliths are bilobate short cells, bulliform, rondel, scutiform-bulliform and square/rectangular. Meanwhile, other morphotypes including hair cell/hair base and irregular multifaceted phytoliths were also extracted; these are found primarily in eudicots leaf based on the available phytolith literature^12,21^. In addition, other phytolith types without taxonomic significance, such as smooth elongate and elongate echinate, are also present. Besides, some sponge spicules were also found in the *Gomphotherium* specimens (Fig. 2L, SI 1.4).

**Table 1:** The counted phytoliths and sponge spicules extracted from *Gomphotherium* molars of two species from the middle Miocene of the Halamagai Formation.

Specimen	Grass phytoliths						Eudicots phytoliths		Unclassed phytoliths	Sponge spicules
	Bilobates short cell	Bulliform	Rondel	Square/ rectanglar	Reed-type bulliform	Long cell	Hair cell/ Hair base	Irregular multifaceted	Elongate	
*Gomphotherium connexum* V8573	5	6	3	10	5	0	15	9	10	7
*G*. *connexum* V8575	4	8	2	11	8	1	19	3	8	13
*G*. *connexum* V8576	13	8	5	12	9	3	22	7	11	10
*G*. *connexum* V18701	1	2	0	3	1	0	3	1	2	1
*G*. *connexum* V8574*	—	—	—	—	—	—	—	—	—	—
*G*. *steinheimense* V23283	25	20	9	15	12	4	0	0	10	5
? *G*. *steinheimense* V24891	3	3	0	2	0	0	0	0	0	0

Note. A total count of 100 phytoliths/sponge spicules was made where possible. Phytolith morphologies are from taxonomies developed by previous studies^12,21^.

^*^Dental calculus are missing in V8574 (*G*. *connexum*) due to severely damaged of the specimen.

Phytolith types exacted from the dental calculus of four studied *G*. *connexum* specimens are consistent. They have relatively high grass phytolith concentration (40–50%). Meanwhile eudicot phytoliths (28–34%) are also consistently present in these four samples (Fig. 2M). Eudicots produce considerably fewer phytoliths on average than grasses^12^, so the presence of eudicot leaf phytoliths very likely indicate that eudicot foliage was a staple dietary component for *G*. *connexum* from our studied area. In other words, our phytolith analysis indicates that *G*. *connexum* was a mixed-feeding generalist that fed on both eudicot foliage and grass, or even primarily a browser.

By contrast, the percentage of grass phytoliths (around 85% including several types of short cells and long cells) in the dental calculus of *G*. *steinheimense* is much higher. Bilobate short cell phytoliths from panicoid grasses (subfamily Panicoideae) are the most common type of short cell^12,21^, comprising around 25% of total phytoliths. Other grass phytoliths include abundant common bulliforms (around 20%) and square/rectangular (around 15%), rondels from pooid grasses^12,21^ (subfamily Pooideae) (around 9%). Long cells (around 4%) were exacted in lesser quantities (Table 1 and Fig. 2M). Large quantities of grass phytoliths among this sample suggest that grass occupied a significant dietary component in the two examined individual of *G*. *steinheimense* from our present study. Moreover, no eudicot phytoliths are present in this sample (Fig. 2M). The result indicates that the *G*. *steinheimense* individuals examined in this study were grazers, rather than browsers.

### Microwear analysis

**Figure 3:**
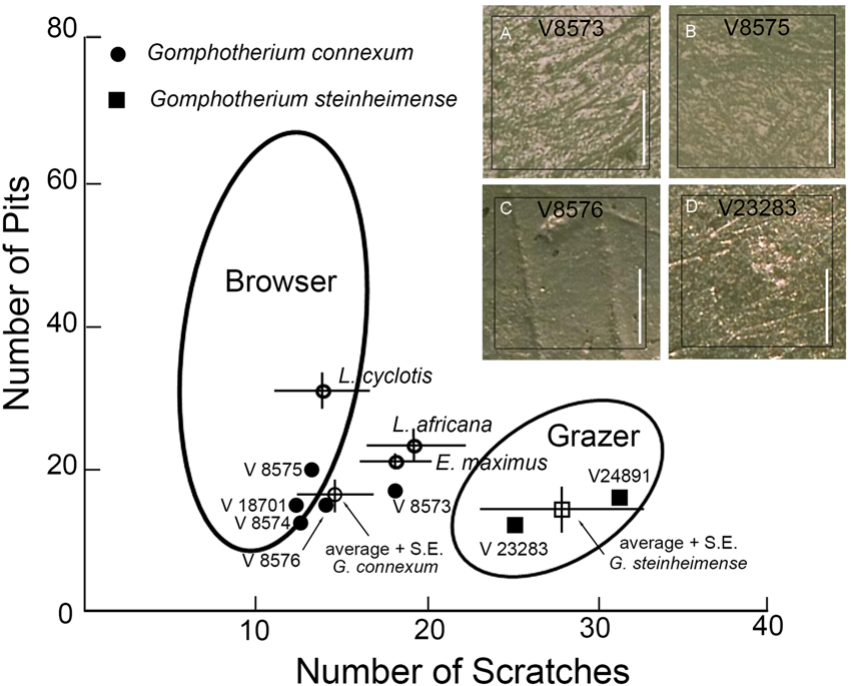
Bivariate plot of the average scratch versus average pit counts in enamel microwear of *G*. *connexum*, *G*. *steinheimense*, and extant elephants (redrawn after ref.^38^). Oval outlines = Gaussian confidence ellipses (p = 0.95) on the centroid of the comparative extant grazer and browser samples adjusted by sample size. The insert panels represent photomicrographs of enamel surfaces of *G*. *connexum* (**A**–**C**) and *G*. *steinheimense* (**D**).

A stereomicrowear analysis of gomphothere dental enamel was performed to further validate the phytolith results. Consistently, the *G*. *connexum* sample displays relatively sparse configuration of thick scratches with scattered pits of irregular size, through micrographs of the molar shearing surfaces (Fig. 3A–C). On the other hand, the *G*. *steinheimense* sample shows dense thin scratches with two (or sometimes more) predominant orientations, which shadow the pits (Fig. 3D). This corroborates the disparity of the feeding habits in the two *Gomphotherium* species indicated by the aforementioned phytolith analyses. The average number of scratches in the studied *G*. *steinheimense* molars are significantly larger than any of the *G*. *connexum* specimens examined in this study (Table S2). The *G*. *steinheimense* samples fall into the area of grazers, whereas *G*. *connexum* from Halamagai is indicated as browser or mixed feeder (Figs 3, S2). This result draws consistent interpretations with respect to the aforementioned phytolith analyses.

### Stable isotope analysis

**Figure 4:**
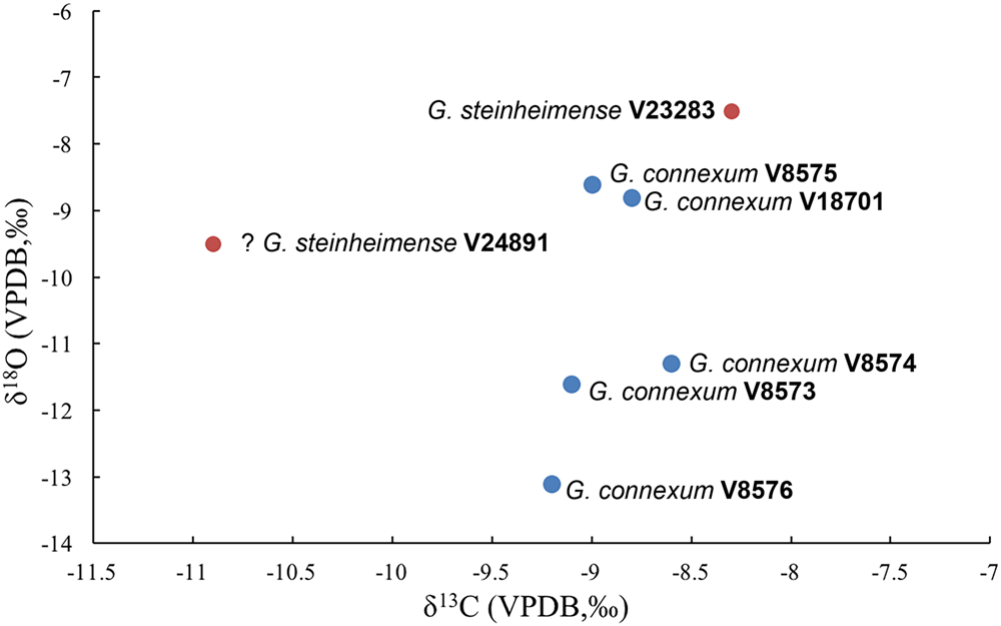
Scatter plot of carbon and oxygen isotope values of the *Gomphotherium* samples.

The δ^13^C values of these six samples range from −10.9‰ to −8.3‰, whereas δ^18^O values are between −13.1‰ and −7.5‰ (Fig. 4, Table S3). Considering the isotopic enrichment (14.1‰) from the diet to bioapatite in large herbivores^6^, the δ^13^C values of all *Gomphotherium* individuals here, averaged by −8.8 ± 0.3‰ (n = 6), show that they consumed mainly C_3_ plants. The relatively small variation of carbon isotope values shows that the *Gomphotherium* species may have had broadly similar habitat preferences, and inhabited a largely homogeneous ecosystem. The relatively high mean δ^13^C value (−8.8‰) of these animals suggests that they probably inhabited a considerably open environment where the C_3_ grasses with higher δ^13^C values were grown^22^. A greater large variability of δ^18^O values, averaged by −10.2 ± 2.2‰ (n = 6), are detected (Fig. 4), suggesting that *G*. *connexum* and *G*. *steinheimense* from the Halamagai Formation may have exploited different sources of water intake, or fed upon plants with different δ^18^O values^22–25^. Nevertheless, considering the fact that the fossiliferous horizon which yielded the *Gomphotherium* fossils here is probably the product of gradual sedimentation over a very long period, during which large climatic fluctuations could have occurred. On the other hand, given the fact that no other animals were used here as isotopic baseline for habitat differentiation, it is not meaningful to discuss the oxygen isotopic variation alone between the two species with unequal sample numbers.

### Cladistic Analysis

A cladistic analysis was performed to investigate the possible phylogenetic relationships of different *Gomphotherium* species. The data matrix contains 52 binary characters and 19 terminals, including three outgroups (Table S1, SI 6.1). Characters 0–49 used follow those of Wang *et al*.^26^, whereas characters 50 and 51 are novel characters incorporated in the present study (Table S1). Cladograms were obtained from a maximum parsimony analysis carried out using the TNT1.1 program with the ‘traditional research’ option^27^. The reported results and node supports were calculated from a 50% majority consensus tree (Fig. 1C).

### Pollen analysis

We analysed a total of 34 pollen samples, among which 21 samples had a relative high pollen concentration, and 13 samples failed to obtain 50 pollen grains or more. A total of 2278 pollen grains were obtained from the 21 samples, 108 in average per sample, consisting of 48 genera and species were counted and identified (Fig. S1 and SI 6.2).

## Discussion

*Gomphotherium* plays an essential role in exploring the evolution of elephantiform proboscideans, as the genus has long been considered to represent the broad distant ancestral stock to more derived elephantiforms, including the extant elephantids or “true elephants”, via the intermediate “tetralophodont gomphothere” grade^4,28^. Tetralophodonts possess at least one more loph(id) on each molar than the trilophodont *Gomphotherium*, an possible adaptation for processing more abrasive foodstuff^4^. Further comparison of enamel stereomicrowear from *G*. *steinheimense* and several tetralophodont gomphotheres (*Tetralophodon xiaolongtanensis*, *Parateralophodon* sp., and *Anancus sinensis*) underpins this hypothesis (Fig. S2). An exception can be found in the stegodontid *Stegolophodon stegodontoides*, which appears to be a mixed feeder (Fig. S2). The stegodontid family are another fully lophodont radiation of proboscideans that originated from the tetralophodonts, in parallel with elephantids^28^. *Stegolophodon* is believed to be ancestral to the Plio-Pleistocene *Stegodon*, both genera characterised by brachyodont (low-crowned) molars^29^, and thought to have fed upon softer foodstuff than the contemporary true elephantids, as a means of niche partition^5^.

Within *Gomphotherium*, our cladistic analysis suggests that *G*. *steinheimense* is the likely sister taxon to *Tetralophodon longirostris*, the archetypical tetralophodont gomphothere. Whereas *G*. *connexum*, the other trilophodont gomphothere from the Halaimagai Formation, is nested as the sister species to the type species *G*. *angustidens* (Fig. 1C). Palaeomagnetic studies indicate that the Halamagai Formation approximately covers the 17–15 Ma age range, corresponding to the Mid-Miocene Climatic Optimum (MMCO)^18–20^ (Fig. 1B and SI 1.1). Subsequently, a decrease of gomphothere diversity took place in northern China during the Late Miocene^30^, which saw the complete replacement of trilophodont gomphotheres, including *Gomphotherium*, by tetralophodont gomphotheres (tri-/tetralophodont replacement event). To a considerable extent, this major turnover has been attributed to the severe aridification of the Asian interior^31^. The Halamagai Formation thus provides a remarkable opportunity to understand the palaeoecological backdrop to this major proboscidean turnover. Our phytolith and microwear results strongly indicate that the two *Gomphotherium* species from the Halamagai Formation show a potential partitioning of feeding ecology. The appreciable presence of eudicot phytoliths from our *G*. *connexum* samples suggests it was an obligate browser or mixed feeder, similar to other *Gomphotherium* species from Europe and North America^32,33^. Whereas more unexpectedly, the two studied specimens of *G*. *steinheimense* may have had a more grass-dominated feeding preference.

The MMCO has previously been detected in deposits from the southern part of the Junggar Basin. Pollen data from the Jingou River section suggests a rapid recovery of regional woodland during 17.3–16.2 Ma^34^. However, our pollen record from the *Gomphotherium*-bearing horizon (the lower part of the Halamagai Formation) reveals that the environment was a grass- and *Artemisia-*dominated prairie accompanied by few arboreal taxa (Fig. S3, SI 2.5, 6.2). Furthermore, in the upper part of the Halamagai Formation and the immediately overlying Kekemaideng Formation, an increase of drought-tolerance shrubs, such as *Ephedra* and *Tamarix*, reveals that prairie had been replaced by a goose-foot-dominated semi-arid savannah (Fig. S3 and SI 6.2). These pollen data thus indicate probable aridity in the northern Junggar Basin during MMCO. This establishment of a grassland-dominated ecosystem may have driven some large herbivores such as *G*. *steinheimense* to modify their feeding behaviour, and become opportunistic to obligate grazers.

The end of the Middle Miocene saw the near-complete extinction of *Gomphotherium* in northern China and the rest of Central Asia^30^, with the exception of *G*. *steinheimense*. Unfortunately, the prerequisite circumstances available for the present study restricts the sample size of *G*. *steinheimense* materials analysed hereby to two specimens, thereby precluding more detailed inferences about the feeding ecology of this species in Middle Miocene Halamagai. However, it is worth noting that *G*. *steinheimense* from the Middle/Late Miocene of Southern Germany has been demonstrated to be a catholic mixed-feeder of both grass and foliage^33^. The modern elephants have long been held as considerably more committed grazer than the gomphotheres, with their molar loph(id)s modified into numerous tightly packed lamellae and considerably higher molar crown (hypsodonty)^4,35^. Nonetheless, empirical studies of elephant feeding behaviour have instead shown the living elephant species as highly generalist megaherbivores, with wild populations typically feeding on over 100 different plant species which span the whole browsing-grazing spectrum, depending on local habitat and seasonality^36^.

As aforementioned, *G*. *steinheimense* quite possibly represents an ancestral lineage to tetralophodont gomphotheres (Fig. 1C), which in turn form the immediate stem grade to elephantids^4,28,36^. Combining this phylogenetic framework with our understanding of past and present proboscidean feeding ecology, it is hereby reasonable to hypothesise that the first evolutionary steps towards the strongly eurytopic feeding ecology seen in modern elephants^36^ was first made by *G*. *steinheimense*, a behavioural tendency which facilitates this species to better exploit a newly abundant food source in the form of grass. On the other hand, more evolutionarily conservative gomphotheres, such as *G*. *connexum*, may have had less flexible feeding preferences to adapt to the significant vegetational turnover in the Middle/Late Miocene, which resulted in the expansion of more open, grassy habitats.

A classic narrative in the evolution of large herbivorous mammals during the Cenozoic is that hypsodonty appeared in various herbivore lineages in response to the spread of grasslands towards the late Neogene, in order to feed on more abrasive vegetation^17,37^. However, Lister^4,37^ noted the prominent temporal lag between a major shift from browsing to grazing in African proboscideans (these include putative direct ancestors of elephantids) (10 Ma) and a significant evolutionary burst towards hypsodonty (5–0.5 Ma). Our Middle Miocene (17–15 Ma) record of a predominantly grazing *Gomphotherium* thus further extends this lag, and supports the hypothesis that within the tetralophodont-elephantid lineage, other modifications to dental morphology (increase in the number of molar loph[id]s and multiplication in the number of conelets on the occlusal facet of each loph[id]) were taking place as a subsequent adaptive response during the lag (Fig. S2)^4,37^. Furthermore, it is worth noting that the fossil record points to the likely origin of tetralophodont gomphotheres in Central Asia at the 18–16 Ma interval^28^, the geochronological context of our study on the feeding ecology of *Gomphotherium* from Halamagai is thus also in immediate correspondence with the origin of the tetralophodont-elephantid lineage, in addition to phylogenetic aspect.

In sum, our present study reveals that the marked Neogene dietary transition from browsing to grazing in the elephantid stem lineage^4,37^ is a process that may be linked back to *G*. *steinheimense* from the MMCO of Central Asia (Fig. S2), at the origin of the tetralophodont-elephantid line. Reconciliation with our current understanding of dietary evolution in elephantimorphs suggests grazing quite probably began through a combination of eurytopic foraging habits^35,36^ and the global expansion of grasslands which began towards the Late Miocene^22,34^, which then led to the suite of grazing-related morphological adaptations which dominated the later evolution and radiation of proboscideans^4,5,28,35,36^. Our study thus further enforces the emergence of grazing in proboscideans and their subsequent adaptive morphological evolution as a crucial case for demonstrating the importance of decoupling the origination of behavioural and phenetic novelties in palaeobiology^4,37^.

## Materials and Methods

### Data availability

The specimens on which this study is based is housed in the collections of the Institute Vertebrate Paleontology and Paleoanthropology (IVPP), Chinese Academy of Sciences. The txt format file for the maximum parsimony analysis (SI 6.1) and xlsx format file for the pollen data (SI 6.2) are available on DRYAD.

### Specimens

Six molars of *Gomphotherium* have been examined, including five specimens of *G*. *connexum* (IVPP V8573–8576, and V18701) (Figs 1E and S4A–C), and one complete m3 of *G*. *steinheimense* (IVPP V23283) (Figs 1D and S4D). Another tooth fragment was referred to as? *G*. *steinheimense* (IVPP V24891), because it displays the diagnostic morphology of *G*. *steinheimense*, details were shown in SI 1.1 (Fig. S4E,F). These specimens are the total *Gomphotherium* sample so far from the study area. Therefore our sample includes most of the available data at the present stage.

### Methods

Dental calculus was carefully removed from the tooth valleys of the study molars, preventing contamination (Figs 2N, S3 and SI 2.1). Phytolith extraction, stereomicrowear analysis, stable isotope study, and pollen extraction were performed using standard methods (SI 2.2, 2.3, 2.4). Cladistic analysis was carried out using TNT1.1 program.

## Electronic supplementary material

Supplementary text

Supplementary Dataset 6.1

Supplementary Dataset 6.2
