# Supplementary material for: A grazing Gomphotherium in Middle Miocene Central Asia, 10 million years prior to the origin of the Elephantidae
Source: Sci Rep. 2018 May 16;8:7640. doi: 10.1038/s41598-018-25909-4 (PMC5956065; doi:10.1038/s41598-018-25909-4)
Supplement: Supplementary file 1 — Supplementary text [file 41598_2018_25909_MOESM1_ESM.docx]

**A grazing *Gomphotherium* in Middle Miocene Central Asia, 10 million years prior to the origin of the Elephantidae**

Yan Wu^1,2^, Tao Deng^1,2,3^, Yaowu Hu^1^, Jiao Ma^1^, Xinying Zhou^1^, Limi Mao^4^, Hanwen Zhang^5,6^, Jie Ye^1^, Shi-Qi Wang^1,2,3^[[1]](#footnote-1)^*^

**Supplementary Information**

**Contents**

1. Supplementary results and discussion

1.1. Additional geological background

1.2. Taxonomic identification of the study material

1.3. Morphological connection between *G. steinheimense* and tetralophodont gomphotheres

1.4. Implication of sponge spicules in dental calculus

2. Detailed methods

2.1. Extraction procedures for fossil samples

2.2. Extraction procedures for phytoliths

2.3. Microwear procedures

2.4. Extraction procedures for isotope sample

2.5. Pollen methods

3. Supplementary references

4. Supplementary figure legends and figures

5. Supplementary tables

6. Supplementary dataset

**1. Supplementary results and discussion**

**1.1 Additional geological background**

The Halamagai Formation is a remarkable site among the Cenozoic deposits from the northern Junggar Basin. Halamagai and the overlying Kekemaideng Formations, characterised by fluviolacustrine green-grayish sandstones and yellow-grayish conglomerates that contain abundant vertebrate fossils, are conspicuously sandwiched into red eolian-dominated mudstones – the underlying Suosuoquan and overlying Dingshanyanchi Formations (Fig. 1B). Palaeomagnetic studies indicate that the base of the Halamagai Formation dates to 16.8 Ma, and the base of the Dingshanyanchi Formation to 13.5 Ma^9^. Therefore, the Halamagai Formation approximately covers the 17–15 Ma, corresponding to the Mid-Miocene Climate Optimum (MMCO)^10^.

The study specimens in the present article were excavated from three fossil sites. IVPP V 8575, 8576, 23283 were from Teersihabahe (46°39’7.84”N, 88°29’47.39”E); V8573, 8574 18701 were from Botamoyin (46°37’43.32”N, 88°20’6.42”E); and V24891 from Teersihabahe East (46°39’37.14”N, 88°30’24.73”E) (Fig. S1). All sites belong to the lower part of the Halamagai Formation. There are considerable distances between these fossil sites (Botamoyin is more than 10 km west to the other two sites). The fluvial sedimentation predominated by pieces of sandstones and conglomerates is unstable, and fossils intersperse among these sandstones and conglomerates. It is difficult to strictly correlate the fossil horizons. In the Teersihabahe and Teersihabahe East sites, there is a remarkable block of mudstones sandwiched in two major sandstones, by which we recognised the upper and lower parts of the Halamagai Formation. In the Botamoyin site, the mudstone layer is almost vanished and we recognised the upper and lower parts only depending on the relative distance to the bottom of the Halamagai Formation.

**1.2. Taxonomic identification of the study material**

In the samples examined, IVPP V8573–V8576 and V18701 have been identified as *G. connexum* (Figs. 1E and S4A–C, also see Wang et al., 2015: fig. 3a, b, e) ^1^.

IVPP V23283 (Figs. 1D and S4D) is a newly diagnosed right m3 that has not been previously published. This specimen was excavated from the same stratus of the Halamagai Formation as the aforementioned *G. connexum* materials. It is large (189.10 × 82.21 mm) and deeply worn with four complete lophids in nearly equivalent width and a week fifth lophid. Complete pretrite trefoils (anterior and posterior central conules, and mesoconelet) are developed on the first three lophids with only slightly larger posterior lobes (posterior pretrite central conules), and the fourth pretrite lophid only has the anterior lobe (the anterior pretrite central conule merged with the pretrite mesoconelet). The fifth lophid is narrow and composed of five subequivalent conelets. Cingulids are developed on the buccal and distal edges, as well as the entoflexid of the first interlophid. The posttrite lophids shows a tendency of transverse elongation and subdivision. It also lacks posttrite central conules. The lophids are crowded and the interlophids are anteroposteriorly compressed relatively to the lower molars of *G*. *connexum* (Fig. S4C). Generally, in *Gomphotherium*, or even in most trilophodont gomphotheres, lophs in upper molars display more crowded and interlophs are more compressed than those in lower molars. Therefore, this feature can only be compared within upper or within lower molars, not between upper and lower molars.

The large dimensions, the nearly symmetric pretrite trefoils, the presence of the fifth lophid, the crowding of lophids and compression of the interlophids, the transverse elongation and subdivision of posttrite half lophids, and the low crown height strongly suggest the similarity to the type specimen of *G. steinheimense* from Steinheim^2^ and other materials described by Göhlich^3^. This is also the first report of *G. steinheimense* from Central Asia.

An additional tooth fragment (Fig. S4E, F) was also attributed to *G. steinheimense*. This piece should be the lateral most part of adjacent two lophs. The two lophs are closely compact with little interloph space, suggesting that the tooth fragment belongs to an upper molar of *G. steinheimense*. The tooth crown is also low as in the typical *G. steinheimense*.

**1.3 Morphological connection between *G. steinheimense* and tetralophodont gomphotheres**

The origin of elephantids (‘true elephants’) from the paraphyletic gomphotheres has remained the consensus among most authorities. Nonetheless, the more precise ancestral candidates for the tetralophodont-elephantid lineage from within the genus *Gomphotherium* have not been particularly well considered. Tassy^6^ discussed that both *G. angustidens* and *G. steinheimense* were the potential candidates. He also delineated *Gomphotherium* species into the ‘*G. annectens* group’ and the ‘*G. angustidens* group’. Wang et al.^7^ further developed the systematics of Tassy^6^. In Wang et al.’s^7^ cladistic analysis, *G. connexum* is nested as the sister taxon to *G. angustidens*, both belonging to ‘*G. annectens* group’, and *G. steinheimense* belongs to ‘derived *Gomphotherium* group’.

*Gomphotherium steinheimense* exhibits closer morphological resemblance to *Tetralophodon longirostris* (the archetypical tetralophodont gomphotheres) than any other taxa of *Gomphotherium* in the following aspects (Fig. S4D–G). 1, *G. steinheimense* possesses circular cross-sectioned mandibular tusks, which is shared with *T*. *longirostris*, and *Stegotetrabelodon*, the earliest elephantid^8^. Circular cross-sectioned mandibular tusks are also present in some members of ‘derived *Gomphotherium* group’, but not in ‘*G. annectens* group’ and ‘*G. angustidens* group’. 2. *G. steinheimense* exhibits relatively closely packed loph(id)s in the molars, which means the anteroposterior compression of interloph(id)s and also probably leads to the transverse elongation of loph(id)s. This feature is also shared with *Tetralophodon longirostris*. 3, The M3s and m3s of *G. steinheimense* are large, the last loph(id)s are wider and more complete than any other *Gomphotherium* species, and the posterior cingulum/cingulid are more prominent, showing a tendency of adding one more loph(id) in the last molars, which suggests an evolutionary transition towards pentalophodonty. To further attest the aforementioned observation, we performed a cladistic analysis. The data matrix is based on Wang et al.^7^, with additional one taxon, *Tetralophodon longirostris*, and two novel characters (see below in Table S1). In the resulting 29 most parsimonious trees (MPTs), *T. longirostris* 100% clusters with *G. steinheimense*, indicating a very strong sister taxa relationship (Fig. 1C).

**1.4 Implication of sponge spicules in dental calculus**

Sponge spicules were identified from the dental calculus of all specimens (except V8574, in which the dental calculus is missing due to severe damage) examined (Table 1). Their presence is significant in indicating the presence of a semiaquatic environment in the habitat of *Gomphotherium*. Thus, both reed-type bulliform phytoliths and sponge spicules occur (Fig. 2L) among our *Gomphotherium* samples and these indicate the presence of an environment abundant in freshwater. The notable disparity in δ^18^O values (Fig. 4) also suggest the two *Gomphotherium* species from Halamagai may have had difference sources of water intake or plant food^9–11^. It may indicate that the studied *Gomphotherium* individuals were feeding and drinking in the vicinity of freshwater.

**2. Detailed methods**

**2.1 Extraction procedures for fossil samples**

Calculus occurs on teeth as a light buff to chocolate brown-stained encrustation above and/or below the gum line. Our sampling process (Fig. S5A) was performed at the IVPP. Standard laboratory gloves were applied and the drilling tools were thoroughly cleaned. Afterwards, specimen surfaces were thoroughly cleaned by blower and acetone before dental calculus could be extracted from the samples (Fig. S5B, C). We extracted samples around the enamel, and limited attention was paid to the nature of a phytolith-containing special structure adhering on the labial surface of the enamel basal portion. Dental calculus was then obtained by scraping and subsequently placed in a tube (Fig. S5D–F). Following this, fresh samples of the special structure were collected within two tubes (one for phytolith analysis and the other for SEM-EDS analysis). Finally, the calculus was then weighted dry.

**2.2 Extraction** **procedures for p****hytolith**

The samples were processed with a procedure slightly modified from Piperno^11^ and Pearsall^12^. The dry powder samples were first treated with 30% H_2_O_2_ solution and heated, followed by 10% HCl solution, then washed by distilled water for three times. The concentration of grains per gram dry sample before mineral separation with a dense solution ZnBr_2_ (d = 2.4). Identification of the phytoliths was conducted using a Nikon Eclipse LV100POL microscope using the keys by Piperno^11^, Pearsall^12^, and IVPP.

For each sample, all morphological parameters were measured at 500 × magnification using a Nikon ECLIPSE LV100 POL light microscope.

**2.3 Microwear procedures**

We observed and analysed microwear under a digital microscope (VHX-600), using the extended depth of focus (EDF) technology. The scratches and pits were quantified in each 0.4 mm × 0.4 mm digital microphotograph^13^. The results were compared to a dataset composed of representative extant proboscidean and ungulate microwear databases^14^. The cast making protocol was based on the ref.^15^.

**2.4 Extraction procedures for isotope sample**

A technique described by Lee-Thorp et al.^16^ was followed to prepare the bioapatite from the tooth enamel. First, contaminants adhering on tooth surfaces were carefully cleaned off and the tooth enamel was then drilled to extract a powdered sample. Then, each of the collected powder samples was individually put in a 1.5 ml centrifuge tube and soaked in 50% sodium hypochlorite (NaOCl) solution for about 48 hours at 4 °C to eliminate organic matter. Once the samples were set up, they were centrifuged at 3200 rpm five times before the supernatants were removed and cleaned with distilled water at least three times to neutralize the pH. Then, 1 mol/L acetic acid solution was added to each sample for about 24 hours to remove any diagenetic carbonate, following the same centrifugation and neutralization procedure as in the third step. Finally, the remains were freeze-dried and ground into powder again.

The carbon and oxygen isotope ratios of enamel bioapatite were measured using Isotope Ratio Mass Spectrometry (Isoprime 100), coupled with a multi-flow system at the Archaeological Stable Isotope Laboratory in the Department of Archaeology and Anthropology, University of Chinese Academy of Sciences. The bioapatite powder of each sample was packed into sealed glass tubes and flushed with high-purity helium. Then a 0.6 ml heated ultrapure phosphoric acid (H_3_PO_4_) solution was injected into every tube using a disposable medical injector at 70^o^C. The reaction was maintained at 80^o^C for an hour to release carbon dioxide, which was eventually transported by helium as carrier gas to IRMS. The standards of IAEA CO-8 and IAEA-603 were used for isotopic calibration. A standard of NBS 18 was inserted into the samples as reference standard for monitoring the measurement stability. The long-term measurement precisions of both δ^13^C and δ^18^O were better than ± 0.2‰.

**2.5 Pollen Method**

For pollen extraction, we followed standard method illustrated by Faegri and Iverson^17^. K/I and ZnI_2_ double heavy liquid was used to extract fossil pollen and spores from the acetolyzed samples^18^.

For each sample, all morphological parameters were measured at 500 × magnification using a Nikon ECLIPSE LV100 POL light microscope.

**3. Supplementary references**

1. Sun, J.-M., Ye, J., Wu, W.-Y., Ni, X.-J., Bi, S.-D., Zhang, Z.-Q., Liu, W.-M. & Meng, J. Late Oligocene–Miocene mid-latitude aridification and wind patterns in the Asian interior. *Geology* **38,** 515–518 (2010).

2. Zachos, J., Pagani, M., Sloan, L., Thomas, E. & Billups, K. Trends, rhythms, and aberrations in global climate 65 Ma to present. *Science* **292,** 686–693 (2001).

3. Wang, S.-Q., Duangkrayom, J. & Yang, X.-W. Occurrence of the *Gomphotherium angustidens* group in China, based on a revision of *Gomphotherium connexum* (Hopwood, 1935) and *Gomphotherium shensiensis* Chang and Zhai, 1978: continental correlation of *Gomphotherium* species across the Palearctic. *Paläontol. Z.* **89,** 1073–1086 (2015).

4. Klähn, H. *Die badischen Mastodonten und ihre süddeutschen Verwandten* (Beil, Berlin, 1922).

5. Göhlich, U. B. Elephantoidea (Proboscidea, Mammalia) aus dem Mittel- und Obermiozän der oberen Süßwassermolasse Süddeutschlands: Odontologie und Osteologie. *Münchner Geowiss. Abh.* **36,** 1–245 (1998).

6. Tassy, P. *La place des mastodontes Miocènes de l’ancien monde dans la phylogénie des Proboscidea (Mammalia): hypothèses et conjecture* (Unpublished Thèse Doctorat ès Sciences, Université Pierre et Marie CURIE, Paris, 1985).

7. Wang, S.-Q., Li, Y., Duangkrayom, J., Yang, X.-W., He, W. & Chen, S.-Q. A new species of *Gomphotherium* (Proboscidea, Mammalia) from China and the evolution of *Gomphotherium* in Eurasia. *J. Vertebr. Paleontol.* **37,** 1–15 (2017).

8. Tassy, P. *in* Fossil Vertebrates of Arabia (eds Whybrow, P. J. & Hill, A.) 209–233 (Yale University Press, New Haven, 1999).

9. Quade, J., Cerling, T. E., Andrews, P. & Alpagut, B. Paleodietary reconstruction of Miocene faunas from Paşalar, Turkey, using stable carbon and oxygen isotopes of fossil tooth enamel. *J. Human Evol.* **28,** 373–384 (1995).

10. Sponheimer, M. & Lee-Thorp, J. A. Oxygen isotopes in enamel carbonate and their ecological significance. *J. Archaeolog. Sci.* **26,** 723–728 (1999).

11. Koch, P. L., Fisher, D. C. & Dettman, D. Oxygen isotope variation in the tusks of extinct proboscideans: a measure of season of death and seasonality. *Geology* **17,** 515–519 (1989).

12. Piperno, D. R. *Phytoliths: A Comprehensive Guide for Archaeologists and Paleoecologists* (Alta Mira Press, Lanham, 2006).

13. Pearsall, D. M. *Paleoethnobotany: A Handbook of Procedures* 2nd edn (Academic Press, San Diego, 2000).

14. Calandra, I., Göhlich, U. B. & Merceron, G. Feeding preferences of *Gomphotherium subtapiroideum* (Proboscidea, Mammalia) from the Miocene of Sandelzhausen (Northern Alpine Foreland Basin, southern Germany) through life and geological time: Evidence from dental microwear analysis. *Paläontol. Z.* **84,** 205–215 (2010).

15. Semprebon, G. M., Deng, T., Hasjanova, J. & Solounias, N. An examination of the dietary habits of *Platybelodon grangeri* from the Linxia Basin of China: Evidence from dental microwear of molar teeth and tusks. *Palaeogeogr. Palaeoclimatol. Palaeoecol.* **457,** 109–116 (2016).

16. Solounias, N. & Moelleken, S. M. C. Tooth microwear analysis of *Eotragus sansaniensis* (Mammalia: Ruminantia), one of the oldest known bovids. *J. Vertebr. Paleontol.* **12,** 113–121 (1992).

17. Lee-Thorp, J. A., Sealy, J. C. & van der Merwe, N. J. Stable carbon isotope ratio differences between bone collagen and bone apatite, and their relationship to diet. *J. Archaeol. Sci.* **16,** 585–599 (1989).

18. Faegri, K. & Iversen, J. *Textbook of Pollen Analysis.* 4th edn (John Wiley & Sons Ltd., Denmark, 1989).

19. Tobien, H. On the evolution of mastodonts (Proboscidea, Mammalia), part 2: The bunodont tetralophodont groups. *Geol. J.b. Hessen* **106,** 159–208 (1978).

**4. Supplementary figure legends and figures**

**Fig. S1 | 3 dimensional reconstruction of topology of the study area, indicating the precise fossil sites and stratigraphic subdivision.** The map was generated by GTOPO309 (http://www1.gsi.go.jp/geowww/globalmap-gsi/gtopo30/gtopo30.html) using Globalmapper (v10) (http://www.bluemarblegeo.com/products/global-mapper.php). The study materials in the present article, IVPP V 8575, 8576, 23283 were from Teersihabahe (46°39’7.84”N, 88°29’47.39”E); V8573, 8574 18701 were from Botamoyin (46°37’43.32”N, 88°20’6.42”E); and V24891 from Teersihabahe East (46°39’37.14”N, 88°30’24.73”E).


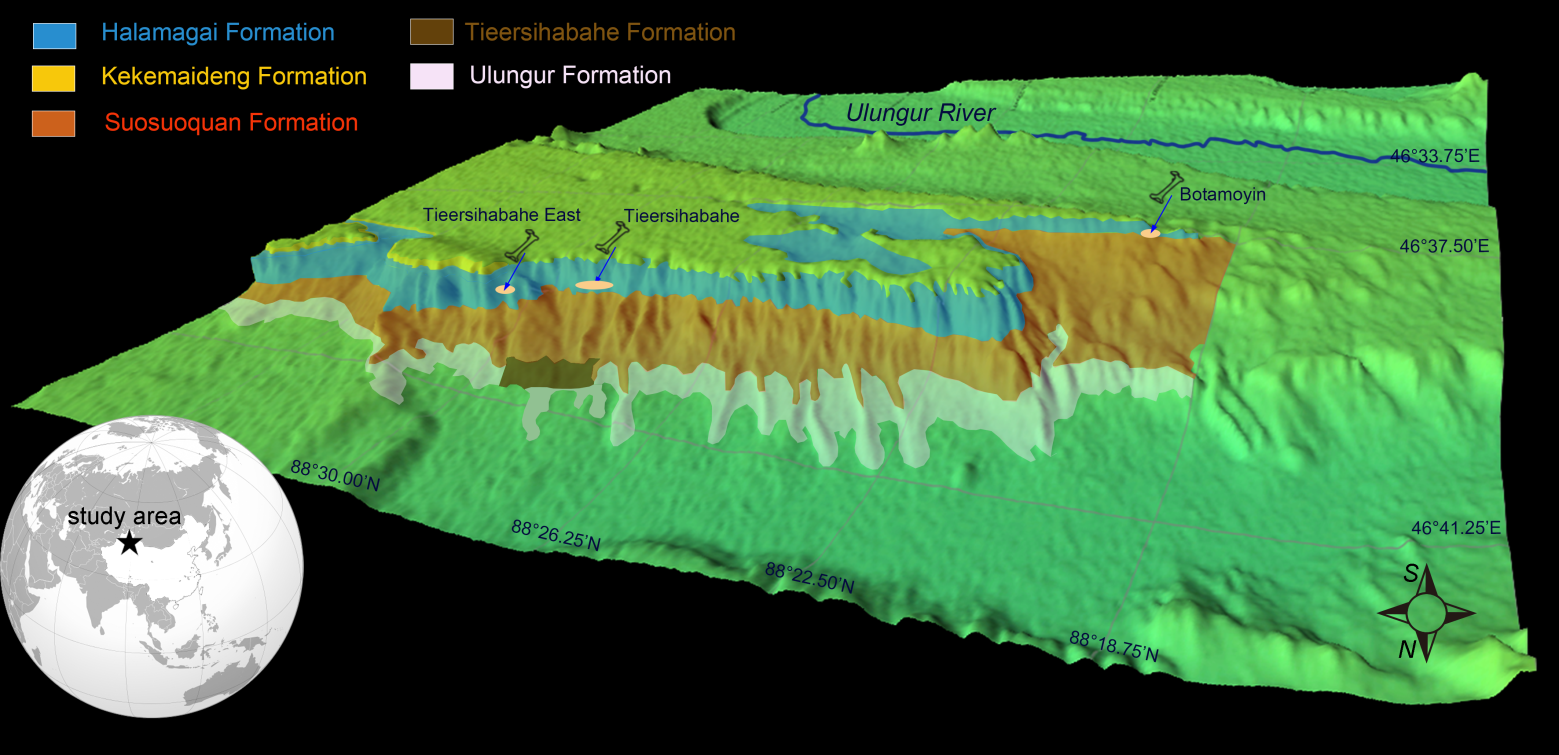


**Fig. S2 | Bivariate plot of the average scratch versus average pit counts in enamel microwear of *Gomphotherium* in the present article and tetralophodont proboscideans (redrawn after ref.^15^).** Oval outlines = Gaussian confidence ellipses (p = 0.95) on the centroid of the comparative extant grazer and browser samples adjusted by sample size. Additional data source: *Tetralophodont xiaolongtanensis*, IVPP V24969, and V24970, from the Xiaolongtan Basin, the latest middle Miocene; *Paratetralophodon* sp., IVPP V3130.4, V3131.1, and V3131.3, from the Lantian area, the early late Miocene; *Anancus sinense*, from the Yushe Basin, the Pliocene; *Stegolophodon stegodontoides*, IVPP V04247, from the Yuanmou Basin, the middle late Miocene.


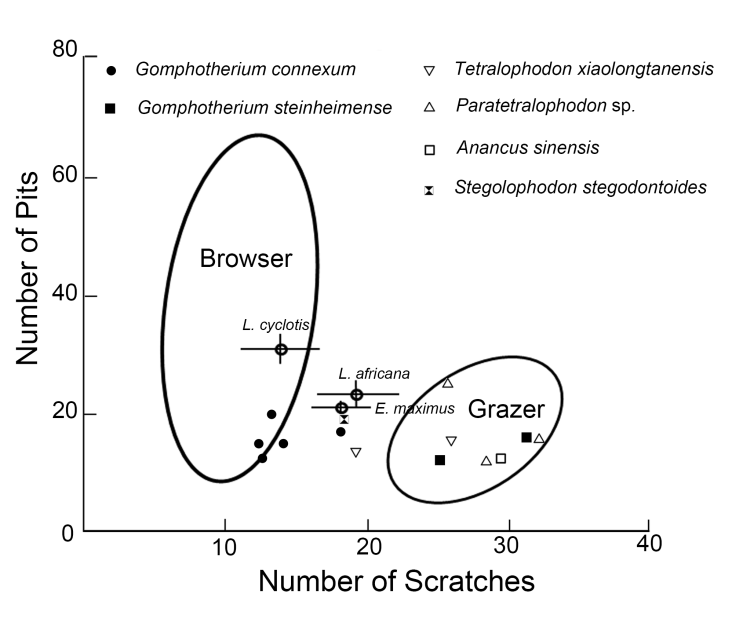


**Fig. S3 | The** **p****ollen percentage spectra of the study area.**


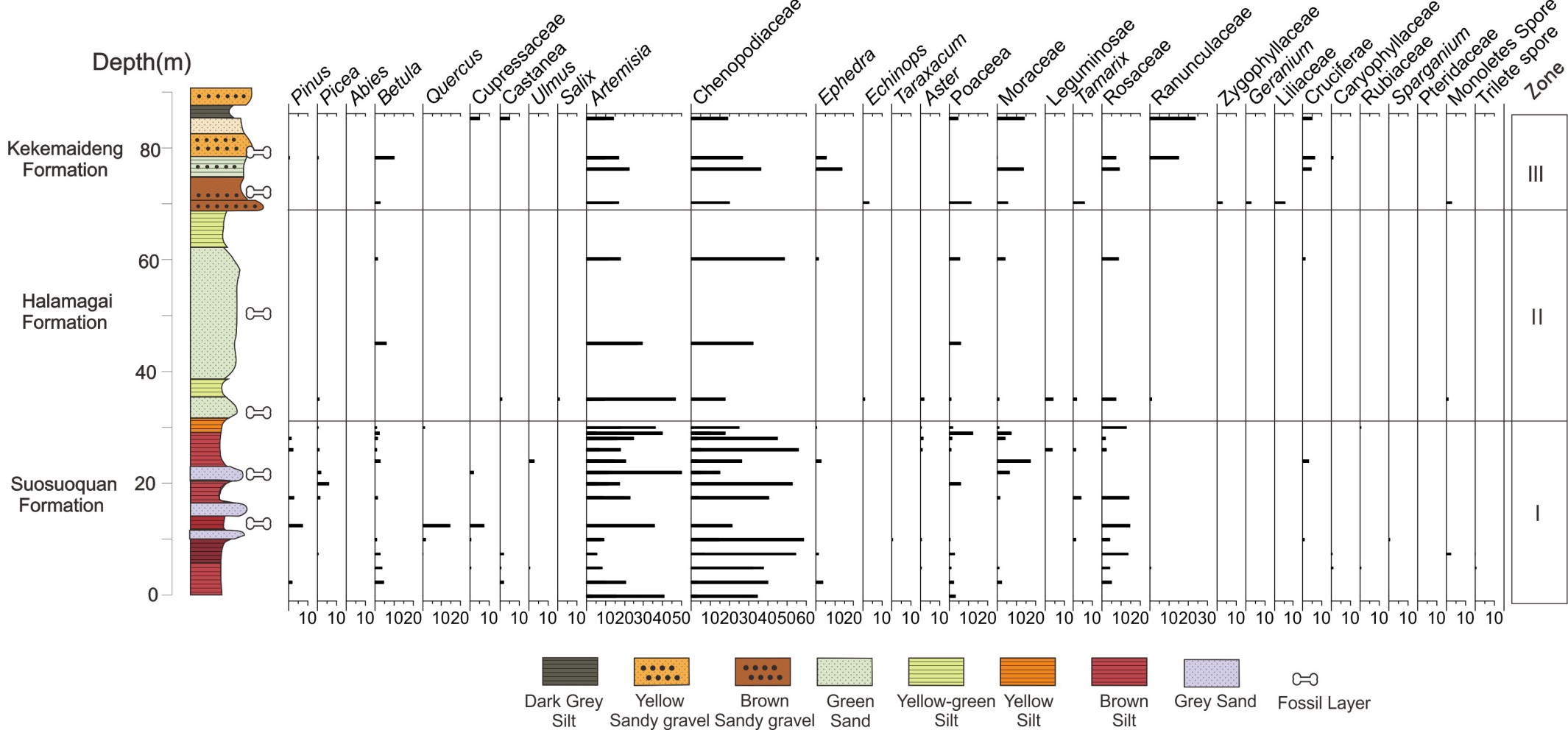


**Fig. S4 | *Gomphotherium* teeth studied in the present paper, and tooth of *Tetralophodon longirostris* for comparison.** **A–C**, *G. connexum*, IVPP V8576 (**A**) and IVPP V8573 (**B**), two left M3s, and IVPP V8575 (**C**), left m3, all from the Halamagai Formation of the Junggar Basin. **D**, *G.* *steinheimense*, IVPP V23283, right m3, from the Halamagai Formation of the Junggar Basin. **E** & **F**, tooth fragment of ? *G.* *steinheimense* IVPP V24891, buccal most part of two posttrite lophs of an upper molar, in occlusal (**E**) and buccal (**F**) views. **G**, *Tetralophodon longirostris*, MNCN 57.615, left M3, from Nombrevilla (Vallesian), Spain. Abbreviations: a-pr-c, anterior pretrite central conule; m-po-c, main posttrite cone; m-pr-c, main pretrite cone; p-pr-c, posterior pretrite central conule; po-meso, posttrite mesoconelet; pr-meso, pretrite mesoconelet.


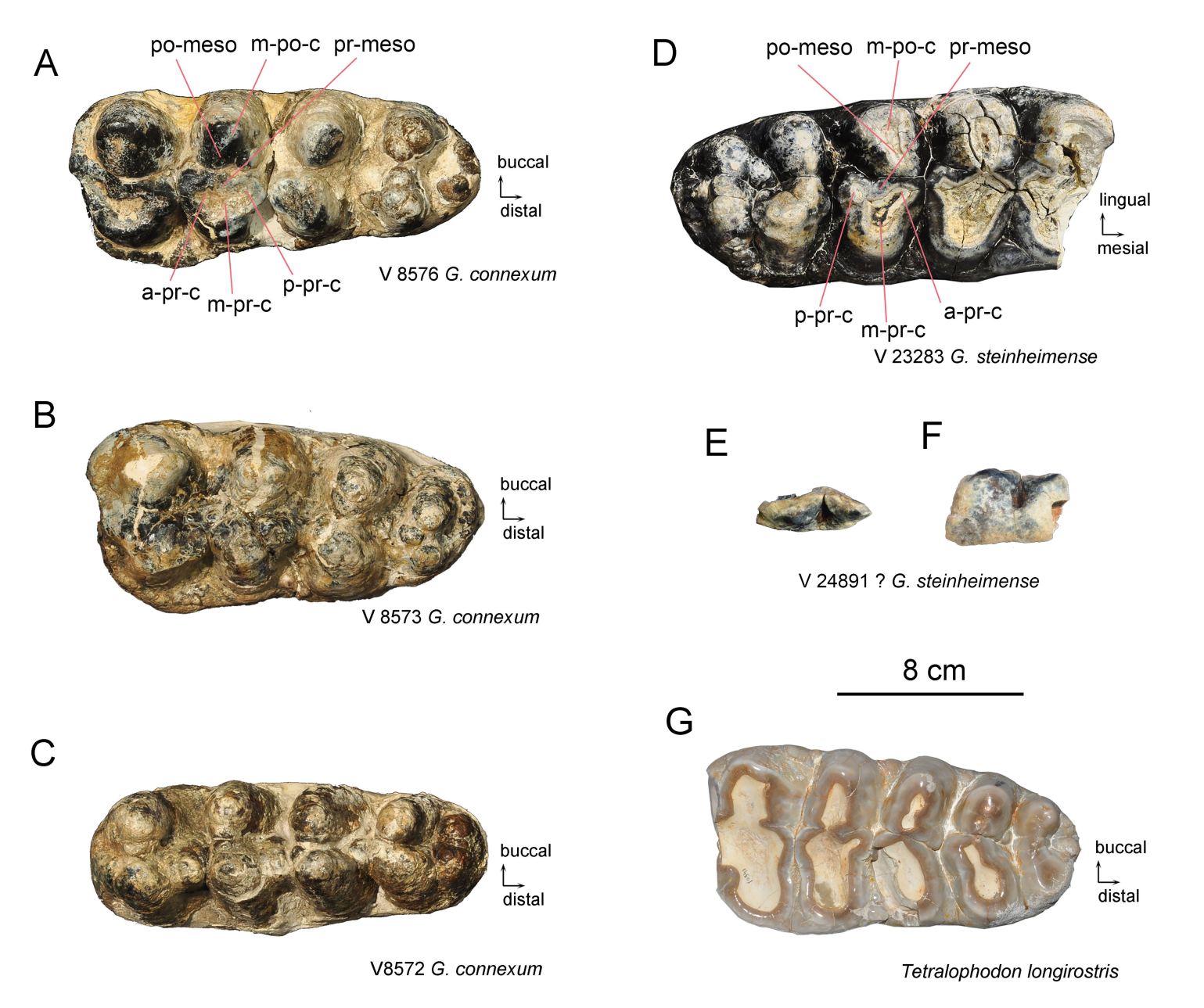


**Fig. S5 | Extraction process for fossil samples.** **A–C**, Surface of the specimen was thoroughly cleaned by blower and acetone. **D–F**, Taking samples and saving them in tubes (the tooth is V8576).


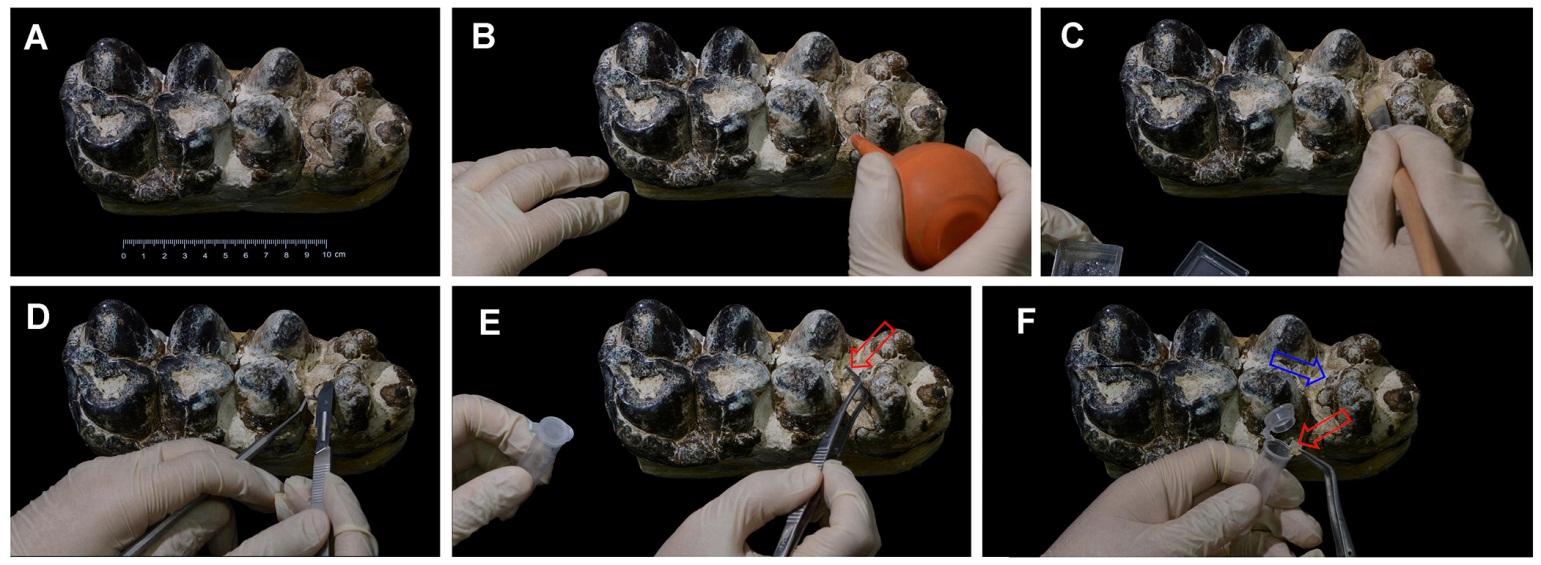


**5. Supplementary tables**

**Table S1. Data matrix in the cladistics analysis**

| Taxon | 0 | 10 | 20 | 30 | 40 | 50 |
| --- | --- | --- | --- | --- | --- | --- |
| *Phiomia* | 0000000000 | 0000000000 | 0000000000 | 0000000000 | 000000??00 | 00 |
| *Eritreum* | ????????00 | ?????10?0? | 000?01???1 | 0??00000?0 | 0000?????? | 00 |
| Mwiti *Gomphotherium* | ?????0???? | ?????????? | 0000?1000? | ?000000?00 | 00000????? | 00 |
| *G. hannibali* | ??00?0???? | ?????????? | 0000?11001 | ?000000000 | 00000????? | 00 |
| *G. annectens* | ??0?010000 | 10???10000 | 0000011001 | 0000000000 | 000000???? | 00 |
| *G. cooperi* | ?????????? | ?????????? | 0000011001 | 0000000100 | 00000????? | 00 |
| *G. sylvaticum* | ?????????? | ??00110010 | ?000111001 | 0000000100 | 0000000000 | 00 |
| *G. libycum* | ?????????? | ?????????? | ?000111001 | 0000100110 | 00000????? | 00 |
| *G. inopinatum* | ???????000 | ?011110001 | ?001111001 | 0000000000 | 00000????? | 00 |
| *G. mongoliense* | ?????????? | ?????10??? | 0001111001 | 0110000010 | 00001????? | 00 |
| *G. angustidens* | 1111111101 | 1111010101 | 1001111101 | 0100101010 | 0000111111 | 00 |
| *G. connexum* | ??????1?01 | ?????1?1?? | 1001111001 | 0101101010 | 00000????? | 00 |
| *G. subtapiroideum* | 0001111000 | 1000110001 | 1101111101 | 0000010100 | 1100001??? | 00 |
| *G. tassyi* | 1111111100 | ?100010001 | 1101111101 | 0000010100 | 11010????? | 00 |
| *G. wimani* | ??????1??? | ?????1???? | 1101111101 | 0000000101 | 11101????? | 00 |
| *G. browni* | ?????11?11 | 1?00111010 | 1001111101 | ?000001011 | 11100????? | 00 |
| *G. steinheimense* | ?????11001 | 0000111010 | 1001111111 | 1000000010 | 11101???10 | 01 |
| *G. productum* | 1100111001 | 0000011000 | 1001111101 | 0000001011 | 110010???? | 00 |
| *T. longirostris* | 0000111001 | 0000111010 | 1001111111 | 1000000011 | 11001????? | 11 |

Note. Characters 0–49 are after Wang et al. (2017: Appendix 1)^7^. Character 50: intermediate cheek teeth. States: 0 = trilophodont; 1 = tetralophodont. Character 51: transverse elongation of loph(id)s. States: 0 = no elongation; 1 = elongated. Source of taxa are also after Wang et al. (2017: Appendix 2)^7^, except *Tetralophodon longirostris* (after ref.^19^)

**Table S2.** **Stereomicrowear results (after ref.^15^) for six *Gomphotherium* molars of two species from the Halamagai Formation**

| **Species** | **Catalog number** | **Scratch** | **Pit** | **SWS** | **Anatomic position** |
| --- | --- | --- | --- | --- | --- |
| *Gomphotherium connexum* | V8573 | 18.1 | 17 | 2 | l. M3 |
|  | V8574 | 12.5 | 12.5 | 3 | l. M3 |
|  | V8575 | 13.3 | 20 | 2 | l. m3 |
|  | V8576 | 13.7 | 15.4 | 3 | l. M3 |
|  | V18701 | 12.4 | 15 | 3 | l. m3 |
| *G.* *steinheimense* | V23283 | 24.5 | 11.83 | 1 | r. m3 |
|  | V24891 | 31.3 | 16 | 1 | ? |

Note. Number of scratch and pit are averaged from three shearing areas for each specimen; SWS = Mean Scratch width score from 0–4 (0 = fine scratches only, to 4 = hypercoarse scratches only)

**Table S3.** **Isotope results for six *Gomphotherium* molars of two species from the Halamagai Formation**

| **Species** | **Catalog number** | **δ^13^C (‰)** | **δ^18^O (‰)** | **Anatomic position** |
| --- | --- | --- | --- | --- |
| *Gomphotherium connexum* | V8573 | -9.1 | -11.6 | l. M3 |
|  | V8574 | -8.6 | -11.3 | l. M3 |
|  | V8575 | -9.0 | -8.6 | l. m3 |
|  | V8576 | -9.2 | -13.1 | l. M3 |
|  | V18701 | -8.8 | -8.8 | l. m3 |
| *G. steinheimense* | V23283 | -8.3 | -7.5 | r. m3 |
|  | V24891 | -10.9 | -9.5 | ? |
| Average (n=7) |  | -9.1 | -10.1 |  |
| standard deviation (n=7) |  | 0.8 | 2.0 |  |

**6. Supplementary dataset**

**6.1 Data set for the maximum parsimony analysis.**

**6.2 Statistic of the pollen data.**

1. ^1^Key Laboratory of Vertebrate Evolution and Human Origins of Chinese Academy of Sciences, Institute of Vertebrate Paleontology and Paleoanthropology, Chinese Academy of Sciences, Beijing 100044, China. ^2^CAS Center for Excellence in Life and Paleoenvironment, Beijing 100044, China. ^3^CAS Center for Excellence in Tibetan Plateau Earth Sciences, Beijing 100101, China. ^4^Key Laboratory of Economic Stratigraphy and Palaeogeography, Nanjing Institute of Geology and Palaeontology, Chinese Academy of Sciences, Nanjing 210008, China. ^5^School of Earth Sciences, University of Bristol, Life Sciences Building, 24 Tyndall Avenue, Bristol BS8 1TQ, UK. ^6^Earth Sciences Department, Natural History Museum, Cromwell Road, London SW7 5BD, UK. *e-mail: wangshiqi@ivpp.ac.cn [↑](#footnote-ref-1)
